# [Corrigendum] Downregulation of miR‑637 promotes proliferation and metastasis by targeting Smad3 in keloids

**DOI:** 10.3892/mmr.2023.13083

**Published:** 2023-09-05

**Authors:** Ye Zhang, Bingyu Guo, Qiang Hui, Wei Li, Peng Chang, Kai Tao

Mol Med Rep 18: 1628–1636, 2018; DOI: 10.3892/mmr.2018.9099

Subsequently to the publication of the above paper, an interested reader drew to the authors’ attention that the Transwell assay data shown in Fig. 4D on p. 1634 contained overlapping sections, such that these data, which were intended to show the results from differently performed experiments, were likely to have been derived from the same original source.

After having examined their original data, the authors have realized that this figure was inadvertently assembled incorrectly. The corrected version of Fig. 4, now showing data in Fig. 4D from one of the repeated experiments, is shown on the next page. Note that this error did not significantly affect the results or the conclusions reported in this paper, and all the authors agree with the publication of this Corrigendum. The authors are grateful to the Editor of *Molecular Medicine Reports* for granting them the opportunity to publish this corrigendum, and apologize to the readership for any inconvenience caused.

## Figures and Tables

**Figure 4.**
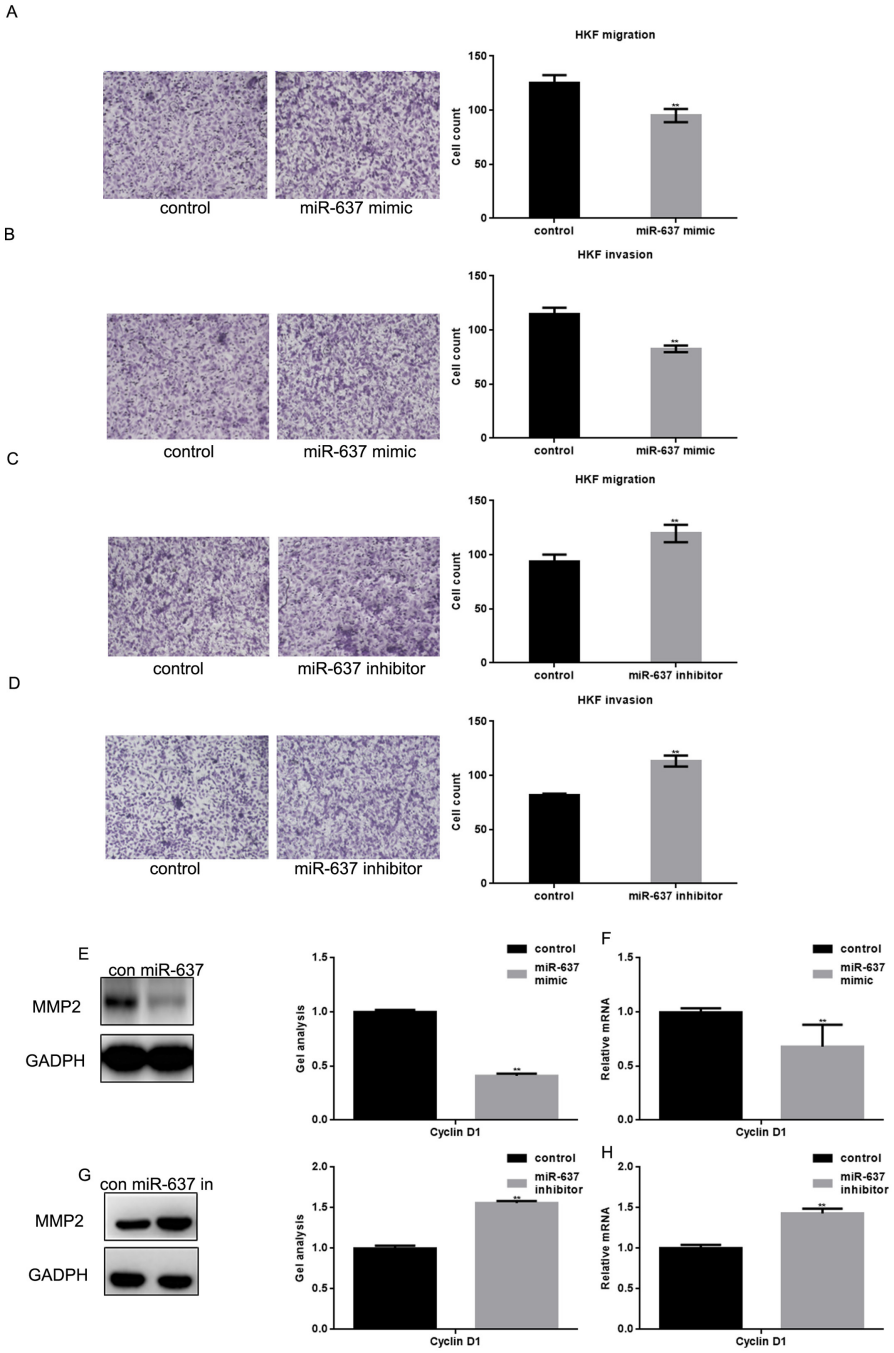
miR-637 inhibits the metastasis of HKF cells by suppressing MMP2. (A and B) After overexpression of miR-637 in HKF cells, transwell assays with or without matrigel were performed. Cells were counted and results represent the mean ± standard deviation of three experiments (magnification, ×200). **P<0.01 vs. control. (C and D) After downregulation of miR-637 in HKF cells, transwell assays with or without matrigel were performed. Cells were counted and results represent the mean ± SD of three experiments. **P<0.01 vs. control. (E and F) After transfection with miR-637 mimic in HKF cells, the expression of MMP2 was detected by western blotting and qPCR. Data are presented as mean ± SEM. **P<0.01 vs. control. (G and H) After downregulation of miR-637, the expression of MMP2 was detected by western blotting and qPCR. Data are presented as mean ± SEM. **P<0.01 vs. control. miRNA, microRNA; qPCR, quantitative polymerase chain reaction; SEM, standard error of the mean; SD, standard deviation; HKF, human keloid fibroblast; MMP, matrix metallopeptidase.

